# Multi-sulfonated ligands on gold nanoparticles as virucidal antiviral for Dengue virus

**DOI:** 10.1038/s41598-020-65892-3

**Published:** 2020-06-03

**Authors:** Antonella Zacheo, Jan Hodek, Dariusz Witt, Giuseppe Felice Mangiatordi, Quy K. Ong, Ozgun Kocabiyik, Nicoletta Depalo, Elisabetta Fanizza, Valentino Laquintana, Nunzio Denora, Danilo Migoni, Piotr Barski, Francesco Stellacci, Jan Weber, Silke Krol

**Affiliations:** 1 Laboratory for nanotechnology, IRCCS Istituto Tumori “Giovanni Paolo II”, Bari, Italy; 2 Institute of Organic Chemistry and Biochemistry of the Czech Academy of Sciences, Prague, Czech Republic; 3 ProChimia Surfaces Sp. z o.o., Sopot, Poland; 4 Istituto di Cristallografia, Consiglio Nazionale delle Ricerche, Bari, Italy; 5 Institute of Materials, Ecole Polytechnique Fédérale de Lausanne (EPFL), Lausanne, Switzerland; 6 Department of Chemistry, University of Bari “Aldo Moro”, Bari, Italy; 7 Institute for Physical and Chemical Processes (IPCF)-CNR, SS Bari, Bari, Italy; 8 Department of Pharmacy - Pharmaceutical Sciences, University of Bari “Aldo Moro”, Bari, Italy; 9 Department of Biological and Environmental Sciences and Technologies (DiSTeBA), University of Salento, Lecce, Italy; 10 Interfaculty Bioengineering Institute, Ecole Polytechnique Fédérale de Lausanne (EPFL), Lausanne, Switzerland; 11 Laboratory for personalized medicine, IRCCS Ospedale Specializzato in Gastroenterologia “Saverio de Bellis”, Castellana Grotte, BA Italy

**Keywords:** Drug discovery, Nanomedicine, Viral infection

## Abstract

Dengue virus (DENV) causes 390 million infections per year. Infections can be asymptomatic or range from mild fever to severe haemorrhagic fever and shock syndrome. Currently, no effective antivirals or safe universal vaccine is available. In the present work we tested different gold nanoparticles (AuNP) coated with ligands ω-terminated with sugars bearing multiple sulfonate groups. We aimed to identify compounds with antiviral properties due to irreversible (virucidal) rather than reversible (virustatic) inhibition. The ligands varied in length, in number of sulfonated groups as well as their spatial orientation induced by the sugar head groups. We identified two candidates, a glucose- and a lactose-based ligand showing a low EC_50_ (effective concentration that inhibit 50% of the viral activity) for DENV-2 inhibition, moderate toxicity and a virucidal effect in hepatocytes with titre reduction of Median Tissue Culture Infectious Dose log_10_TCID_50_ 2.5 and 3.1. Molecular docking simulations complemented the experimental findings suggesting a molecular rationale behind the binding between sulfonated head groups and DENV-2 envelope protein.

## Introduction

Dengue virus (DENV) belongs to the family Flaviviridae which are usually transmitted by mosquitos or ticks and are responsible for a variety of human diseases mainly haemorrhagic fevers (Dengue, yellow fever, West Nile) but also encephalitis and jaundice and lately Zika^1^. According to the WHO 4 serotypes of Dengue exist DENV1–4. The risk of dengue infections is now present in 128 countries affecting almost half of the world population and resulting in 390 million of infections per year^2,3^. Infections lead to diseases with large variety of severity ranging from asymptomatic to mild dengue fever and to severe dengue haemorrhagic fever and dengue shock syndrome with about 500,000 people yearly requiring hospitalization^4,5^. Currently, there is no effective antiviral or safe universal vaccine for DENV infections. The only available vaccine (DENGVAXIA), recently approved by FDA, presents high risk to unexposed individuals and is therefore administered to people with laboratory-confirmed previous dengue infection^4^.

The continuous increase of DENV infections in endemic areas as well as the lack of efficient countermeasures underline the need for new therapeutics. There are different routes to interrupt the DENV replication cycle: (i) inhibiting intracellular targets such as two of the main DENV enzymes, namely protease^6–8^ and RNA-dependent RNA polymerase^9–18^ or (ii) structural glycoprotein envelope (E) protein of the DENV^4,19^. Inhibiting the entry step is an attractive approach to prevent viral infections^20^. The search for DENV entry inhibitors focused on the main three domains on the E protein, the stem domain, hydrophobic pocket in the hinge domain and the receptor binding domain^19^. One of the most potent compounds, compound 6, targets the hydrophobic pocket of the E protein, and hence blocks all four DENV subtypes with sub-micromolar potency^20^ Other peptide inhibitors aim at the stem and receptor binding domain resulting in disruption of attachment, entry and fusion process^21^.

Recently different types of nanoparticles (NP) have been proposed as antiviral treatments. NP can be used as drug delivery system for traditional antiviral drugs to increase their efficacy and reduce side-effects or the NP themselves are the drug. An overview summarizing metal NP as antivirals can be found in Rai *et al*.^22^. Drug-loaded NP mainly aim at intracellular inhibition mechanisms and can be administered either i.v. injection for a systemic treatment of Dengue, or as an oral application for the manifestation in the gastrointestinal system and the liver which is usually indicative for a more severe, often life-threatening form of Dengue^23^. However, the nanoparticulated intracellular drug delivery faces mainly the same obstacles as the drug alone. Using the NP as the drug itself, therefore, seems more promising.

In particular, it has been shown that a hybrid surface coating containing silver, copper and zinc cation led to a reduction of DENV-2 replication of 80% and log_10_TCID_50_ DENV-2 titre reduction of 1.1^24^. Murugan *et al*. showed that silver NP had significant virus-inhibitory properties^25,26^. Another study described gold NP (AuNP) coated with mercapto-undecansulfonic acid (MUS) ligands that mimic the heparan sulfate proteoglycans (HSPG) binding site on the cell surface^27^. The attachment of HSPG-binding viruses such as herpesvirus, HIV, papilloma, or Dengue virus to the NP surface successfully blocked infection in mammalian cells. The authors showed that the virucidal mechanism was based on strong multivalent binding of ligands and dependent on their specific length, which induced an interaction with the HSPG-binding viruses so strong that it led to local distortions. This resulted in global virus deformations and eventually in the irreversible loss of infectivity, *i.e*. virucidal mechanism. The concept of HSPG binding was also used by Dey *et al*.^28^. who introduced a nanogel with sulfonate groups as a broad spectrum antiviral.

In this study we varied the chain length as well as the number of sulfonate groups presented by one ligand as well as different sugars as head group presenting the sulfonate groups in different orientations to the virus in order to understand the factors influencing the nanoparticle-virus interaction. We found that a multi-sulfonated complex ligand with a glucose headgroup was somewhat more efficient than the linear mono-sulfonated ligand MUS which was used in previous experiments. This compound showed promising potential as antiviral drug as it induced a virucidal effect inhibiting most of the virus permanently. Molecular docking simulations, performed on the ligand-binding pocket of the DENV-2 envelope protein, suggest the presence of key interactions between the sulfonate substituents of the head groups and specific residues of the protein, hence providing a molecular rationale behind the antiviral activity of the ligands.

## Results and Discussion

To reduce the EC_50_ of sulfonated AuNPs, induce a virucidal rather than virustatic effect, and achieve a different spatial presentation of the sulfonated groups, we chose six different ligands with multiple sulfonate groups as ligands for a self-assembling monolayer on AuNP (L-AuNP). The results of the new L-AuNPs were compared with previously tested MUS-AuNPs^27^.

### Nanoparticle preparation and characterization

To evaluate whether the synthesis of the L-AuNP was achieved with all ligands, we performed TEM analysis. We obtained L-AuNP with all ligands except for L3 (Fig. 3). Additional to the modified Stucky we tried even ligand-exchange with L3 on citrate-coated AuNP to get L3-coated AuNPs but particles after overnight incubation were solved.

To verify the presence of L1, L2, L4, L5 and L6 at the AuNP surface, we visualized the organic ligands using a TEM negative staining procedure. For all samples, we observed grey halos surrounding the AuNP that could be clearly distinguished from the darker background and represent the ligands (Supplementary Fig. S9). For L4-AuNP, the halo was fainter than for the other samples, probably owing to the shorter chain length of L4 (Supplementary Fig. S9c).

In addition, we confirmed the presence of the fluorescent L1 on the AuNP surface using UV-Vis spectroscopy (Supplementary Fig. S7). In three independent L1-AuNP samples, we observed a broad absorbance peak at 528 nm corresponding to the localized surface plasmon resonance (LSPR) band of the AuNP, which was also observed for MUS-AuNP. We also detected a distinct absorbance peak at 466 nm that corresponds to L1. The absence of free ligands in the NP solution after washing was confirmed by NMR measurements (exemplarily for L6-2 in Supplementary Fig. S6).

**Figure 1:**
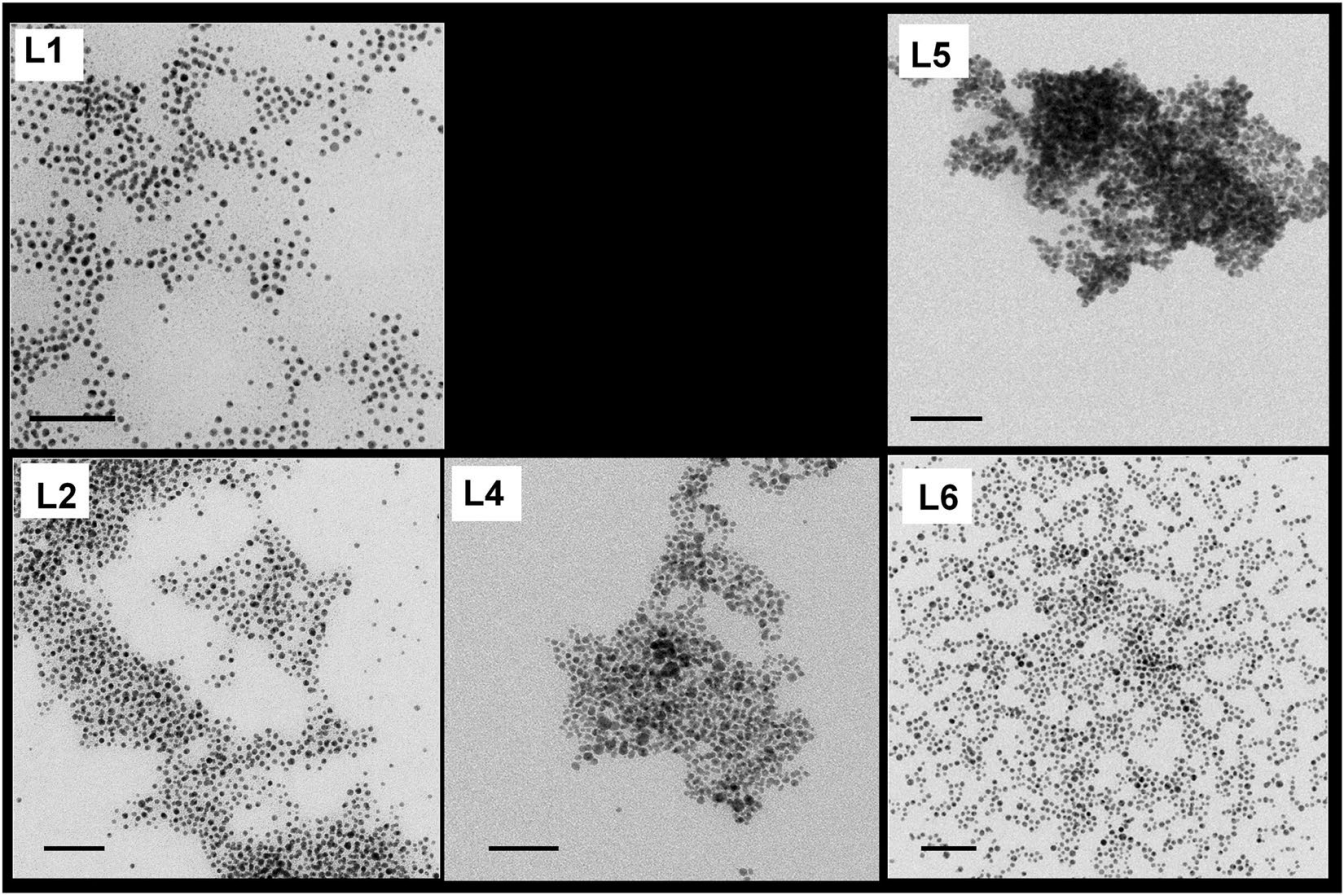
Transmission electron micrographs of AuNP prepared by Stucky method in presence of Ligand 1–6. Scale bar 50 nm.

The particles coated by L1, 2, 4–6 were characterized by DLS and TEM (Fig. 1) for their appearance, diameter, and size distribution and by ZP for the NP surface charge (Table 1). For each type of sample, the evaluation of the average size and size distribution of AuNP was achieved by performing the statistical analysis (ImageJ) of the corresponding TEM micrographs. As it can be seen in Table 1, the size of the AuNP of around 4–5 nm is comparable for all prepared particles. The size distribution bar graph can be found in Supplementary Fig. S8. For L5 AuNP, the statistical analysis was not possible as the particles were mainly aggregated as it can be observed in the corresponding TEM micrograph (Fig. 1,L5).

**Table 1:** Physicochemical properties of the multi-sulfonated ligand coated AuNP (L-AuNP) and mono-sulfonated ligand coated AuNP (MUS-AuNP).

Compound^a^	Chain length [CH_2_]_x_	No. of sulfonates ^per^ ligand molecule	Head group	Core size^b,c^ [nm]	Hydrodynamic diameter by number [nm]^d^ (PDI)	Zeta potential [mV]
MUS-AuNP	11	1	—	4.3 ± 1.3*	18.9 ± 3.4 (0.364)	−38 ± 5.3*
L1-AuNP n = 5	11	3	pyridine	4.0 ± 1.8; N = 295	8.7 ± 1 (0.30275)	−37.2 ± 9
L2-AuNP n = 4	11	8	glucose	4.1 ± 3.3; N = 788	76.5 ± 6.1 (0.1922)	−39.6 ± 3.2
L4-AuNP n = 3	2	8	glucose	4.2 ± 2.1; N = 502	46.5 ± 19.4 (0.3994)	−17.6 ± 1.2
L5-AuNP n = 2	11	12	lactose	n.d.	81.3 ± 28.2 (0.2876)	−42.2 ± 2.6
L6-AuNP n = 2	11	12	maltose	4.7 ± 1.7; N = 698	25.4 ± 7.4 (0.229)	−41.9 ± 6

^a^n: number of independent experiments. ^b^determined by TEM; average ± std dev.; ^c^N: number of analysed particles; ^d^determined by DLS by particle number average ± std dev (n = 3–5) (PDI = polydispersity index). *Data from Verma *et al*.^36^.

AuNP surface, that can be certainly ascribed to the sulfonate groups of the ligands (Fig. 3, Table 1), thus suggesting MUS and L1-L6 act as NP capping agents binding the NP surface through the thiol or disulphide or cleaved disulphide groups.

The presence of ligands at NP surface was also evidenced by TEM micrographs obtained by staining, as the staining procedure induce a contrast and visualize the organic ligand on NP surface. Indeed, in Fig. S9 reported in SM, representative TEM images of AuNP synthesized in presence of L1, 2 and 4 were shown. Similar results were achieved for AuNP capped with L5 and 6, having a chemical structure similar to that one L2. The presence of the organic shell at NP surface can be clearly observed as grey halo surrounding the AuNP, which appears darker than background. For L4 capped AuNP, the organic shell resulted less evident, probably owing to the shorter length of the chains in the chemical structure of L4 respect to the other ligands (Supplementary Fig. S9C).

### Anti-dengue activity of nanoparticles

To evaluate the anti-dengue activity of the different L-AuNP, we measured both their cytotoxic concentration that reduces the target cell viability by 50% (CC_50_) and their effective concentration that inhibits 50% of the viral activity (EC50; Table 2). Each L-AuNP was incubated with DENV-2 for 1 hour before added to recipient cell, Vero and HepG2-hNTCP. The CC_50_ and EC_50_ values were determined 3 days after infection.

**Table 2:** Anti-dengue activity of the L-AuNP.

Compound	Chain length [CH_2_]_x_	No. of sulfonates per ligand molecule	Head group	CC_50_ [µg/mL]	95% CI^a^ of CC_50_	EC_50_ [µg/mL]	95% CI^a^ of EC_50_	SI^b^
**Vero cells**								
MUS-AuNP	11	1	—	>10	n.a.	10.5	6.9–16.1	>1
L1–3-AuNP	11	3	pyridine	75.1	68.1–83.0	33.9	24.5–46.9	2.2
L2–3-AuNP	11	8	glucose	78.7	63.6–97.4	4.3	3.3–5.5	18.3
L4-1-AuNP	2	8	glucose	333	270–410	27.6	19.8–38.4	12.1
L5-1-AuNP	11	12	lactose	54	48.1–60.6	80.8	60.5–108	<1
L5-2-AuNP				83.3	65.3–106	118	85.8–163	<1
L6-1-AuNP	11	12	maltose	34.9	17.7–68.7	62.3	45.3–85.8	<1
L6-2-AuNP				50.9	40.8–63.5	44.4	30–65.6	1.1
**HepG2-hNTCP cells**								
L2-3-AuNP	11	8	glucose	148	139–158	15.7	12.1–20.3	9.4
L6-2-AuNP	11	12	maltose	268	252–286	12.7	8.34–19.2	21.1

^a^CI:95% confidence interval; ^b^SI: selectivity index; L5-1-AuNP, L5-2-AuNP, L6-1-AuNP, and L6-2-Au-NP are independent preparations of L5-AuNP, and L6-AuNP.

We observed the highest Vero cell toxicity for L6-1-AuNP, and the lowest for L4-1-AuNP. However, we found no clear trend in toxicity dependent on the number of sulfonate groups per ligand molecule or the chain length of the molecule. The presence of free ligands in the nanoparticle solutions that can be toxic was excluded by NMR analysis of the L-AuNP solutions (Supplementary Fig. S6). The multi-sulfonated L2-AuNP was more efficient at inhibiting DENV-2 than the linear long-chain mono-sulfonated ligand MUS, which could indicate that the spatial orientation and the number of sulfonate groups influences the binding to the viral envelope. The toxic effect of L2–3-AuNP and L6-2-AuNP on HepG2-hNTCP cells was about two to five time lower than in Vero cells. The low hepatocellular toxicity of L6-2-AuNP resulted in best selectivity index of all tested L-AuNP. The difference between CC_50_ in Vero and HepG2-hNTCP cells could be probably explained by different uptake of AuNP by kidney vs. liver cells. The nanoparticles can behave differently than classical chemical compounds and it is possible that nanoparticle size, shape, charge and density influence the uptake in kidney differently than in liver.

To determine whether the effect of the L-AuNP on the virus was reversible or permanent, i.e. whether the L-AuNP were virustatic or virucidal, respectively, we performed a virucidal assay. We first incubated the L-AuNP with DENV-2 followed by serially diluting the mixtures and adding them to both types of recipient cells.

We found that only L2- and L6-AuNP showed a virucidal effect while the binding was reversible for all other L-AuNP (Table 3). Because during DENV infection the liver is particularly affected^29^, we decided to evaluate the antiviral and virucidal potential of multi-sulfonated ligand-coated AuNP in hepatocytes. We selected L2-3-AuNP that exhibited best EC_50_ and L6-2-AuNP that showed best virucidal effect in Vero cells. Both L2-3-AuNP and L6-2-AuNP showed good virucidal activity and 2.5 to 3.1 log_10_TCID_50_ DENV-2 titre reduction for L2-3-AuNP and L6-2-AuNP, respectively. These results are in agreement with the importance of heparan sulfate proteoglycan during the entry of DENV-2 into hepatocytes^30^. We are aware of discord between antiviral and virucidal results, especially with the case of L6-AuNP in Vero cells. To achieve strong virucidal effect by AuNPs it is necessary that large amount of AuNPs is attached to the viral particle. Only multivalent binding of the AuNPs to the viral particle leads to irreversible viral damage as demonstrated by TEM analysis of AuNP interaction with Herpes simplex virus 2 by Cagno *et al*.^27^. In virucidal experiment in Vero cells, we have combined very high concentration of L6-AuNP directly with the virus and possibly achieved strong multivalent binding of L6-AuNPs to the DENV-2 leading to more irreversible damage. Indeed, in the L6-2-AuNP virucidal experiment we have used ten times higher concentration than in L2-3-AuNP virucidal experiment. In accord, using almost two times lower amount of L6-1-AuNP (different L6-AuNP preparation) resulted in 2.3 log_10_TCID_50_ lower titer reduction in comparison with L6-2-AuNP. In the antiviral experiment, we have combined range of different L6-AuNPs concentrations with the same amount of DENV-2 and probably did not achieve optimal amount of AuNP that would inhibit virus without toxicity to the Vero cells. Thus, resulting in no difference between EC_50_ and CC_50_ for L6-AuNPs. Further research will be necessary to elucidate optimal stoichiometry of viral particles and AuNPs.

**Table 3:** Virucidal assay of the L-AuNP to determine if the ligand shell induces an irreversible loss of infectivity.

Ligand	Virus titer reduction expressed in log_10_TCID_50_ (or as percentage)	
	DENV-2 infection in Vero cells	DENV-2 infection in HepG2hNTCP cells
L1–3-AuNP	0	n.d.
L2–3-AuNP	0.6 (73%)	2.5 (99.7%)
L4-1-AuNP	0	n.d.
L5-1-AuNP	0	n.d.
L5-2-AuNP	0	n.d.
L6-1-AuNP	2.6 (99.8%)	n.d
L6-2-AuNP	4.9 (100%)	3.1 (99.9%)

n.d. not determined; L5-1-AuNP, L5-2-AuNP, L6-1-AuNP, and L6-2-AuNP are independent preparations of L5-AuNP, and L6-AuNP.

### Molecular docking

To identify whether the sulfonated headgroups determine anti-dengue activity of the L-AuNP, we performed molecular docking simulations of three sugar head groups, namely sulfonated glucose (L2-head), lactose (L5-head) and maltose (L6-head). We assumed that the CH_2_-chains did not contribute to the binding because they are bound to the AuNP surface. In our docking simulations, we used a previously published X-ray structure of the β-OG binding pocket of the DENV-2 E protein as starting protein structure (PDB code. 1OKE)^31,32^.

**Figure 2:**
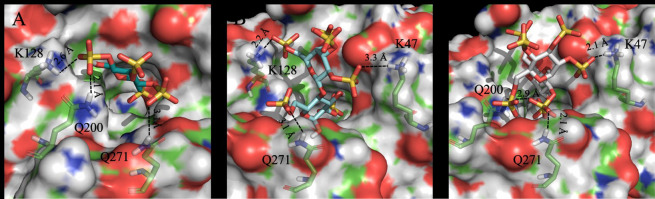
Top-scored docking poses of the L2-head (**A**), L5-head (**B**) and L6-head (**C**) within the binding site of the DENV-2 E protein (PDB code: 1OKE). For the sake of clarity only polar hydrogens are shown. Important residues were rendered as sticks while the protein was represented as a surface. Hydrogen bonds and salt-bridge interactions are depicted by dotted lines.

Figure 2 shows the top-scored poses obtained for the three sulfonated sugar head groups, glucose, lactose and maltose. The L2 achieved the best docking score (30.11 kJ/mol) while L5 and L6 obtained similar score of 22.59 kJ/mol and 22.58 kJ/mol, respectively. Interestingly, all considered ligands establish H-bond interactions with the amino acid Q271in the binding site of DENV-2, already proven to be crucial for ligand binding of β-OG^33^. Although the protein pocket is characterized by many basic residues, potentially able to interact with sulfonated groups, the best docking score is obtained by the head group of L2 provided with the lowest number of sulfonate groups but properly oriented to establish strong salt-bridge and H-bond interactions with the amino acids K128, Q200 and Q271. Many sulfonated groups of the L5- and L6-heads were not able to interact with the protein pocket. It is worth to note that the pocket cavity was strongly hydrophobic except for a small solvent-exposed part responsible for the accommodation of the sugar head of the ligands. The extended polar portion of the molecule of L5 and L6 may have been forced, at least partially, into the hydrophobic part of the cavity thus leading to a lower binding energy.

The docking scores for the L2- and L6-heads were in good agreement with the experimental data, but the docking scores for the L5-head differed significantly from the experimental data. This difference could be explained by the aggregation of L5-AuNP and their bigger size, which imposes a steric hindrance to the head group. This could prevent strong binding of the L5-head to the binding pocket of the virus.

In the present study, we compared the virus inhibition and virucidal effect of small colloidal AuNP coated by five different multi-sulfonated complex sugar ligands (L-AuNP) with those of AuNP coated with a linear mono-sulfonated ligand (MUS-AuNP) previously reported as virus entry inhibitor^27^. The aim was to determine if spatial distribution of the sulfonate groups, chain length of the linker between gold surface and multi-sulfonated head groups or number of sulfonated head groups has an influence on the antiviral properties especially with focus on developing virucidal AuNP.

We found that L2 consisting of a disulphide with two C11 alkyl chains and a tetra-sulfonate glucose headgroup was most efficient in inhibiting DENV-2 and showed a virucidal effect. The reason for this was likely that it fits better in the β-OG pocket on the virus as we confirmed by docking experiments and therefore can induce stronger forces and hence irreversibly damage the virus. We determined that the L2-head yielded the highest docking score to the β-OG pocket on the E envelope protein of DENV-2. For L5 and L6, characterized by multi-sulfonated lactose and maltose headgroups, respectively, we determined similar and somewhat lower docking scores. However, the ligands differed in their efficiency to inhibit DENV-2; L5 performed significantly worse than L6, likely because of the aggregation of the AuNP.

There seems to be no direct correlation of the inhibitory efficiency to the number of sulfonated groups but to their spatial presentation to the virus and longer linker work better than very short ones. This is in good agreement with the findings by Cagno *et al*.^27^.

Recently Dighe *et al*.^32^ summarized EC_50_ and IC_50_ values for potential anti-dengue drugs as part of extensive drug screening projects or ongoing clinical trial. The values reported by them are in the same range or higher than the EC_50_ values found by us. We identified multi-sulfonated ligand coated AuNP as new antivirals for DENV-2. The low toxicity profile and the virucidal rather than virustatic interaction with the DENV-2 underlines their potential as candidate for in-depth studies in future *in vivo* experiments.

## Methods

### Ligand preparation and characterization

**Figure 3:**
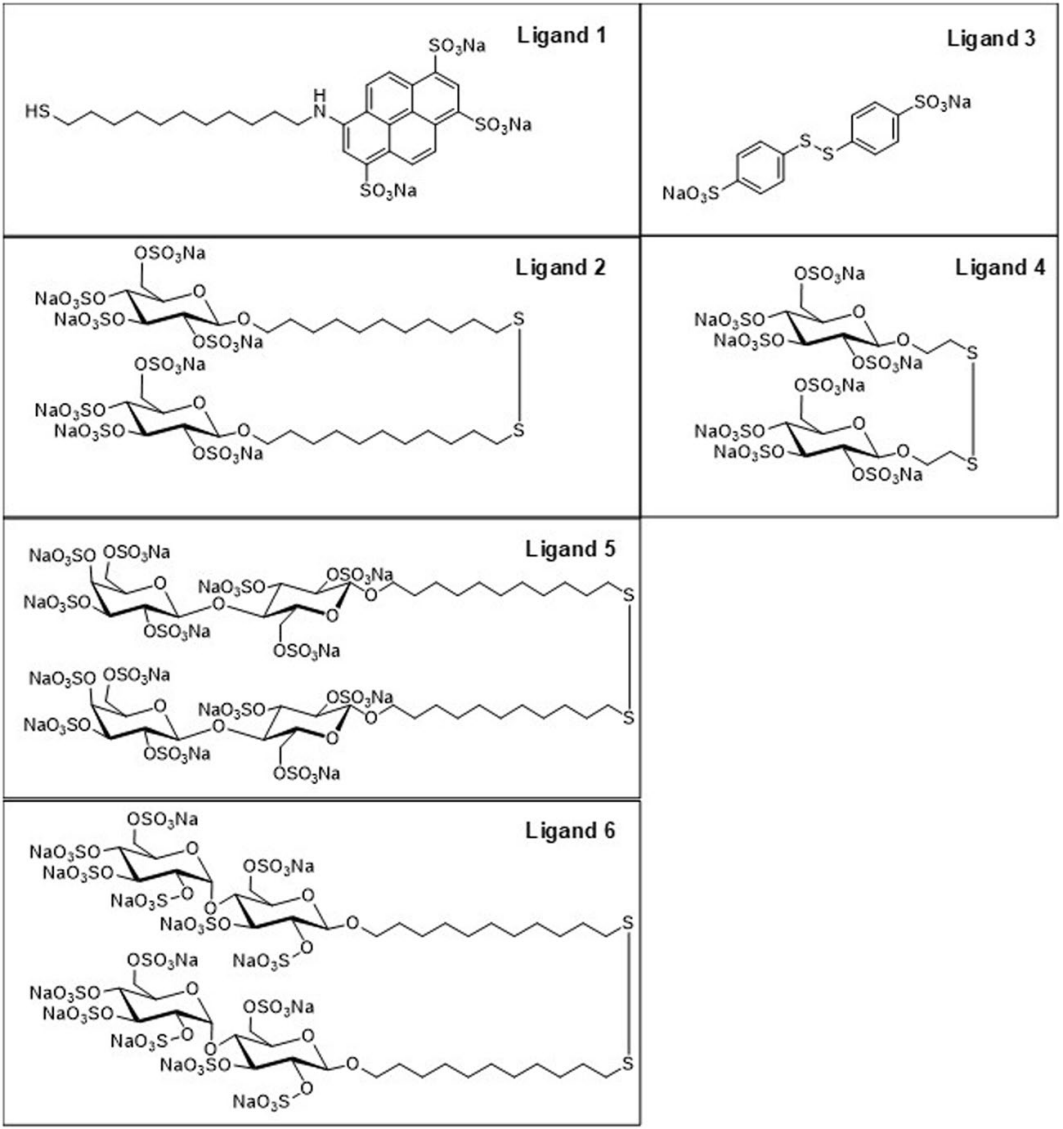
Chemical structure of ligands L1-L6.

The mono-sulfonated ligand MUS (cat. No.: FT 009) was provided by Prochimia, Poland. The multi-sulfonated ligands, shown in Fig. 3, are (a) trisodium 8-(*N*-11-mercaptoundec-1-ylamino)pyrene-1,3,6-trisulfonate (**Ligand 1 or L1**); (b) Bis-11-(2,3,4,6-tetra-*O*-sodium sulfonato-β-D-glucopyranosyl)undec-1-yl disulfane (**Ligand 2 or L2;** cat. no. DI 009, Prochimia, Poland); (c) Disodium 4,4’-disulfanediyldibenzenesulfonate (**Ligand 3 or L3;** cat. no. CH 004-m11, Prochimia, Poland); d) Bis-2-(2,3,4,6-tetra-*O*-sodium sulfonato-β-D-glucopyranosyl)ethan-1-yl disulfane (**Ligand 4 or L4;** cat. no. CH 004-m2, Prochimia, Poland); e) Bis-11-(2,3,4,6-tetra-O-sodium sulfonato-β-D-galactopyranosyl-(1 → 4)-2,3,6-tri-*O*-sodium sulfonato-β-D-glucopyranosyl)undec-1-yl disulfane named **Ligand 5 or L5 (**cat. no. CH 006, Prochimia, Poland) and f) Bis-11-(2,3,4,6-tetra-O-sodium sulfonato-α-D-glucopyranosyl-(1 → 4)-2,3,6-tri-*O*-sodium sulfonato-β-D-glucopyranosyl)undec-1-yl disulfane (**Ligand 6 or L6;** cat. no. CH 008, Prochimia, Poland)**)**, were designed, synthesized and characterized by Prochimia Surfaces (Poland). L3 was synthesized according to a protocol published by Smith *et al*.^34^. To assess whether the ligands L1, 2, 4–6 were synthesized successfully, ^1^H Nuclear magnetic resonance (NMR) spectroscopy was performed (Supplementary Figs. 1–5).

### Nanoparticle preparation

AuNP were synthesized coated with L1–6 (L-AuNP) using a modified Stucky procedure^35^ previously described by Stellacci and co-workers^36–38^. Briefly, 0.13 mmol of chloro(triphenylphosphine)gold(I) was dissolved in 46 mL of a 9:1 dimethylformamide (DMF) to deionized water mixture in a 100 mL round bottom flask at room temperature (RT). Three different flasks were prepared. In each of them, the respective sulfonated ligand dissolved in a 5 mL mixture of a 1:1 DMF:H_2_O mixture was added (0.03 mmol L1; 0.015 mmol L2, L4–6) and stirred for few minutes. 25 mg of borane tert-butylamine complex, previously dissolved in a 5 mL mixture of DMF:H_2_O 1:1, was quickly added to the reaction mixture and the flask was connected to a condenser. The reaction was carried out at 120 °C under reflux for 2 h 30 min. After this, the samples were cooled at RT and the AuNP were purified by repeated salting out. Finally, the samples were washed 5 times with DI-water by centrifugal ultrafiltration (Vivaspin 20, Sartorius; 10 kD NMWL). We succeeded in preparing AuNP with all ligands except L3.

After preparation, the L1-, L2-, L4-, L5- and L6-AuNP were either lyophilized and stored as a powder until further use or washed and used as solutions. Each preparation was performed at least in duplicate. For each ligand, several batches of nanoparticles were prepared. In the following, we will name the ligand Lx-y, x being the ligand and y being the batch identifier.

### Nanoparticle characterization

#### Dynamic light scattering and zeta potential

The physicochemical properties of the ligand coated AuNP (mean hydrodynamic diameter, polydispersity index and surface charge) were evaluated by dynamic light scattering (DLS) and zeta potential (ZP) measurements on a Zetasizer Nano ZS (Malvern Instruments Ltd., Worcestershire, UK). Measurements were performed at 25 °C. For the hydrodynamic diameter and size distribution measurement by DLS, the NP stock solution was measured. The ZP was determined by laser doppler velocimetry of the stock solution in water. Each sample was measured three times: the reported values correspond to the averages of these values and the reported errors to the standard deviation.

#### Transmission Electron Microscopy

A JEOL med. 100 electron microscope operating at 100 kV equipped with a charge-coupled device high resolution camera was used for TEM analysis. Samples were prepared by directly applying several microliters of sample onto carbon coated copper grids and dried prior to imaging. The core size of the AuNP was determined by image analysis using FIJI (ImageJ). Specimen staining for TEM observation, was achieved, after the sample deposition, by covering the grid with a small drop (5 µL) of an aqueous phosphotungstic acid (Sigma-Aldrich) solution 2% (w/v). After 30 sec, the drop was removed; finally, the grid was washed with ultrapure water and dried at room temperature.

#### Inductively Coupled Plasma Atomic Emission Spectroscopy

The amount of gold was determined by Inductively Coupled Plasma Atomic Emission Spectroscopy (ICP-AES) and the calculated concentration used for the efficacy study and toxicity study. The samples were first mixed with 4 mL of H_2_O_2_ and 6 mL of superpure HNO_3_ 69%, then treated at 180 °C for 10 min using a microwave digestion system (Milestone START D). Afterwards, the samples were cooled, diluted with ultrapure water to a final volume of 20 mL, filtered through syringe filters (pore size: 0.45 μm), and measured for gold content using an ICP-AES (Thermo Scientific, iCap 6000 Series) spectrometer. The spectrometer was previously calibrated for quantitative analysis with four standard solutions containing known concentrations of gold: 0.001, 0.01, 0.1, and 1.0 mg/L. The calibration line showed a correlation coefficient (r) greater than 0.99 for the measured element. The results were the average of three different measurements, and the gold final concentrations were expressed as mg/L.

#### UV-Vis spectrometry

To compare size and polydispersity qualitatively, we measured UV-Vis spectra of all AuNP were measured at 20 °C with a Perkin-Elmer Spectrometer Lamba Bio20, equipped with a 10 mm quartz cuvette.

### Anti-Dengue activity and toxicity

DENV-2 (strain 16681) was obtained from Dr. Jochen Bodem, University of Wurzburg (Wurzburg, Germany). Vero cells were obtained from the European Collection of Cell Cultures (Salisbury, UK) and maintained in Dulbecco’s Modified Eagle’s Medium (DMEM) with L-glutamine, 10% foetal bovine serum, 100 U of penicillin/mL and 100 μg of streptomycin/mL (all Sigma-Aldrich, St. Louis, USA) in 5% CO_2_ at 37 °C. HepG2-hNTCP (human liver cancer cells stably transduced with human sodium taurocholate co-transporting polypeptide^39^) cells were obtained from Dr. Stephan Urban, Heidelberg University Hospital (Heidelberg, Germany) and were maintained in DMEM prepared as above and supplemented with 0.05 mg/mL of puromycin (Sigma-Aldrich). Both cell lines were mycoplasma negative (tested at Generi Biotech, Czech Republic).

Vero cells were chosen as the cell line known to be susceptible for DENV-2 infection while the HepG2-hNTCP as a human hepatic cell culture model^39^. For the anti-dengue activity of the L-AuNP we determined the effective concentration that inhibits 50% of the viral activity (EC_50_), and the cytotoxic concentration that reduces the target cell viability by 50% (CC_50_. We also determined whether the effect on the virus was virustatic (indicating a reversible inhibition) or virucidal (indicating an irreversible inhibition).

For the EC_50_ test, two-fold dilutions of L-AuNP were mixed with DENV-2 and incubated for 1 h at RT. Then the mixture was added to 20,000 Vero cells or 30,000 HepG2-hNTCP cells in triplicate to achieve a multiplicity of infection of 0.3 IU/cell. After 72 h incubation, the anti-dengue activity was determined by an immuno-fluorescent assay as described in ref. ^24^. Fluorescent microscopy images were processed by the NIH ImageJ program^40^. Drug concentrations required to reduce fluorescence by 50% (EC_50_) were calculated using nonlinear regression analysis with the GraphPad Prism version 8.2.1 for Windows (GraphPad Software, La Jolla, USA). For the CC_50_ test, the experimental conditions were the same as for the EC_50_ experiments. The cytotoxicity of the NP was evaluated by a colorimetric Cell Proliferation Kit II (XTT; Sigma-Aldrich) in a Victor X3 plate reader (Perkin Elmer, Waltham, USA) and the CC_50_ values were calculated as the EC50 values. Selectivity indexes were calculated as ratio CC_50_/EC_50._

For the virucidal assay in Vero cells, 10 µl DENV-2 (22762 IU/mL titre for L1–3, L2–3, L4-1 80954 IU/mL titre for L5-1, L6-2) was mixed with 10 µl NP (1.97 mg/mL for L1–3, 0.85 mg/mL for L2–3, 1.66 mg/mL for L4-1, 4.72 mg/mL for L5-1, 10.62 mg/mL for L5-2, 4.80 mg/mL for L6-1, and 8.84 mg/mL for L6-2) and incubated for 1–2 hours (2 hours for L1–3, L2-3, L4-1 and 1 hour for L5-1, L5-2, L6-1 and L6-2) at room temperature. For the virucidal experiment in HepG2-hNTCP, we used same batch of DENV-2 as for virucidal experiments with L5-AuNP and L6-AuNP, but titre in hepatocytes was determined as 1,151,675 IU/ml (more than 10 times higher than titre in Vero cells). The titre is determined during the particular virucidal experiment from control experiment where we used the same amount of DENV-2 not exposed to AuNP and treated identically as mixtures with AuNP. Then, the mixture was two-fold diluted (12 dilutions in total) and each dilution was added to 20,000 Vero cells or 30,000 HepG2-hNTCP in triplicate. After 3 days of incubation in 5% CO_2_ and 37 °C, an immunofluorescence assay was performed as described above. Plates were analysed with an Olympus fluorescence microscope (Carl Zeiss, Jena, Germany) and wells with at least 10 red loci were considered positive. Virus titres were calculated using the Reed-Muench method^41^ and expressed as infectious units per mL. Titre reduction was calculated as the difference between titres from untreated and treated virions and expressed in percentage.

### Molecular docking

The sulfonated sugar head groups (glucose, lactose and maltose) were subjected to docking simulations conducted on the crystal structure of the DENV-2 envelope protein in complex with n-octyl-beta-D-glucoside (β-OG) (PDB code: 1OKE^33^). GOLD (Genetic Optimization for Ligand Docking^42^) was used as software for both, protein preparation and flexible ligand docking. More specifically, the protein structure was prepared using default setting for adding missing hydrogen atoms, assigning protonation states and predicting the histidine tautomers. A spherical grid having a radius of 10 Å centred on the centre of mass of the cognate ligand was used. All the default flexible ligand docking settings and the fitness function CHEMPLP^43^ were employed. An exception is represented by the starting population size, increased to a value equal to 1000 in order to properly explore the conformational space of the ligands within the binding site during the simulation. The original X-ray cognate ligand was redocked back into its corresponding protein binding site. In particular, a RMSD value equal to 1.99 Å was obtained after comparing the Cartesian coordinates of the corresponding heavy atoms of the obtained pose thus supporting the robustness of the applied docking protocol.

## Supplementary information

Supplementary Information.
