# Supplementary material for: Multi-sulfonated ligands on gold nanoparticles as virucidal antiviral for Dengue virus
Source: Sci Rep. 2020 Jun 3;10:9052. doi: 10.1038/s41598-020-65892-3 (PMC7271158; doi:10.1038/s41598-020-65892-3)
Supplement: Supplementary file 1 — Supplementary Information. [file 41598_2020_65892_MOESM1_ESM.docx]

**Electronic supplementary material**

**Multi-sulfonated ligands on gold nanoparticles as virucidal antiviral for Dengue virus**

Antonella Zacheo^1^, Jan Hodek^2^, Dariusz Witt^3^, Giuseppe Felice Mangiatordi^4^, Quy K Ong^5^, Ozgun Kocabiyik^5^, Nicoletta Depalo^6^, Elisabetta Fanizza^6,7^, Valentino Laquintana^8^, Nunzio Denora^7,8^, Danilo Migoni^9^, Piotr Barski^3^, Francesco Stellacci^5,10^ , Jan Weber^2^, Silke Krol^11,#^

^1^ Laboratory for nanotechnology, IRCCS Istituto Tumori “Giovanni Paolo II”, Bari, Italy

^2^ Institute of Organic Chemistry and Biochemistry of the Czech Academy of Sciences, Prague, Czech Republic. Mail: weber@uochb.cas.cz

^3^ ProChimia Surfaces Sp. z o.o., Sopot, Poland

^4^ Istituto di Cristallografia, Consiglio Nazionale delle Ricerche, Bari, Italy;

^5^ Institute of Materials, Ecole Polytechnique Fédérale de Lausanne (EPFL), Lausanne, Switzerland

^6^ Department of Chemistry, University of Bari "Aldo Moro", Bari, Italy

^7^ Institute for Physical and Chemical Processes (IPCF)-CNR, SS Bari, Bari, Italy

^8^ Department of Pharmacy - Pharmaceutical Sciences, University of Bari "Aldo Moro", Bari, Italy

^9^ Department of Biological and Environmental Sciences and Technologies (DiSTeBA), University of Salento, Lecce, Italy

^10^ Interfaculty Bioengineering Institute, Ecole Polytechnique Fédérale de Lausanne (EPFL), Lausanne, Switzerland

^11^ Laboratory for personalized medicine, IRCCS Ospedale Specializzato in Gastroenterologia "Saverio de Bellis", [Castellana Grotte (BA)](https://www.bing.com/local?lid=YN1354x9166423506832051203&id=YN1354x9166423506832051203&q=IRCCS+De+Bellis&name=IRCCS+De+Bellis&cp=40.88395309448242~17.155357360839844&ppois=40.88395309448242_17.155357360839844_IRCCS+De+Bellis&FORM=SNAPST#_blank), Italy

**Figure S1**. ^1^H NMR spectrum of L1. ^1^H NMR (500 MHz, methanol-d4) δ = 9.29 (s, 1H, Ar), 9.14 (d, J = 9.7 Hz, 1H, Ar), 9.05 (d, J = 9.7 Hz, 1H, Ar), 8.88 (d, J = 9.8 Hz, 1H, Ar), 8.48 (d, J = 9.8 Hz, 1H, Ar), 8.13 (s, 1H, Ar).3.54 (t, J = 7.1 Hz, 2H, CH2-N), 2.48 (t, J = 7.1 Hz, 2H, S-CH2), 1.95-1.80 (m, 2H, CH2), 1.60-1.20 (m, 16H, CH2)

(**A**)

(**B**)**Figure S2**. ^1^H NMR spectrum of L2. (**A**) ^1^H NMR (500 MHz, methanol-d4) δ = 4.25 (d, J = 7.8 Hz, 2H, beta-H, Glu), 3.95-3.84 (m, 4H, Glu), 3.70-3.64 (m, 2H, Glu), 3,58-3.51 (m, 2H, Glu), 3.38-3.24 (m, 6H, Glu, OCH_2_-), 3.20-3.14 (m, 2H, Glu), 2.69 (t, J = 7.3 Hz, 4H, S-CH_2_-), 1.78-1.55 (m, 8H, CH_2_), 1.50-1.20 (m, 28H, CH_2_) (**B**) 1H NMR (500 MHz, D_2_O) δ = 4.50-3.20 (m, 18H, Glu, OCH_2_), 2.50-2.70 (m, 4H, S-CH_2_-), 1.70-1.40 (m, 8H, CH_2_), 1.40-1.00 (m, 28H, CH_2_)

**Figure S3**. ^1^H NMR spectrum of L4. ^1^H NMR (500 MHz, D_2_O) δ = 4.90-3.80 (m, 18H, Glu, OCH_2_), 2.90 (t, J = 7.1 Hz, 4H, S-CH_2_-)

**Figure S4**. ^1^H NMR spectrum of L5. ^1^H NMR (500 MHz, D_2_O) δ = 5.20-3.40 (m, 32H, Gal, Glu, OCH2), 2.60 (t, J = 7 Hz, 4H, S-CH_2_-), 1.70-1.40 (m, 8H, CH_2_), 1.40-1.00 (m, 28H, CH_2_)

**Figure S5**. ^1^H NMR spectrum of L6. ^1^H NMR (500 MHz, D_2_O) δ = 5.50-3.40 (m, 32H, Glu, OCH_2_), 2.60 (t, J = 7 Hz, 4H, S-CH_2_-), 1.70-1.40 (m, 8H, CH_2_), 1.40-1.00 (m, 28H, CH_2_)


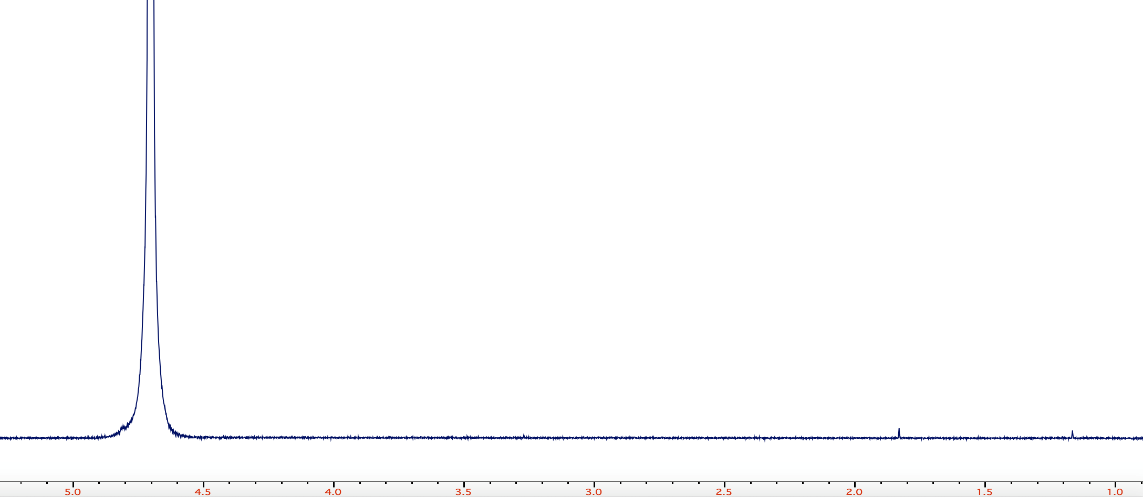


**Figure S6**.^1^H NMR spectrum of AuNPs coated by a ligand shell of L6-2 after purification. No impurities by unbound ligands can be observed.


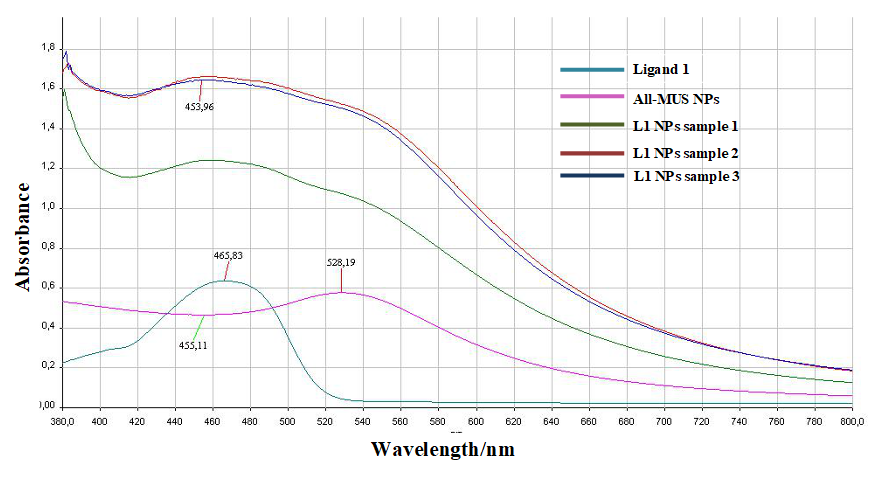
**Figure S7**. UV-Vis measurement of the Ligand 1 and three L1 AuNP samples prepared in independent experiments. The all MUS coated AuNPs without absorbing ligand present the absorbance spectrum of AuNPs of approximately the same size. UV-Vis spectra were measured at 20°C with a Perkin-Elmer Spectrometer Lamba Bio20, equipped with 10mm quartz cell.

**Figure S8**. Nanoparticle core size and size distribution determined by ImageJ analysis of the TEM images.


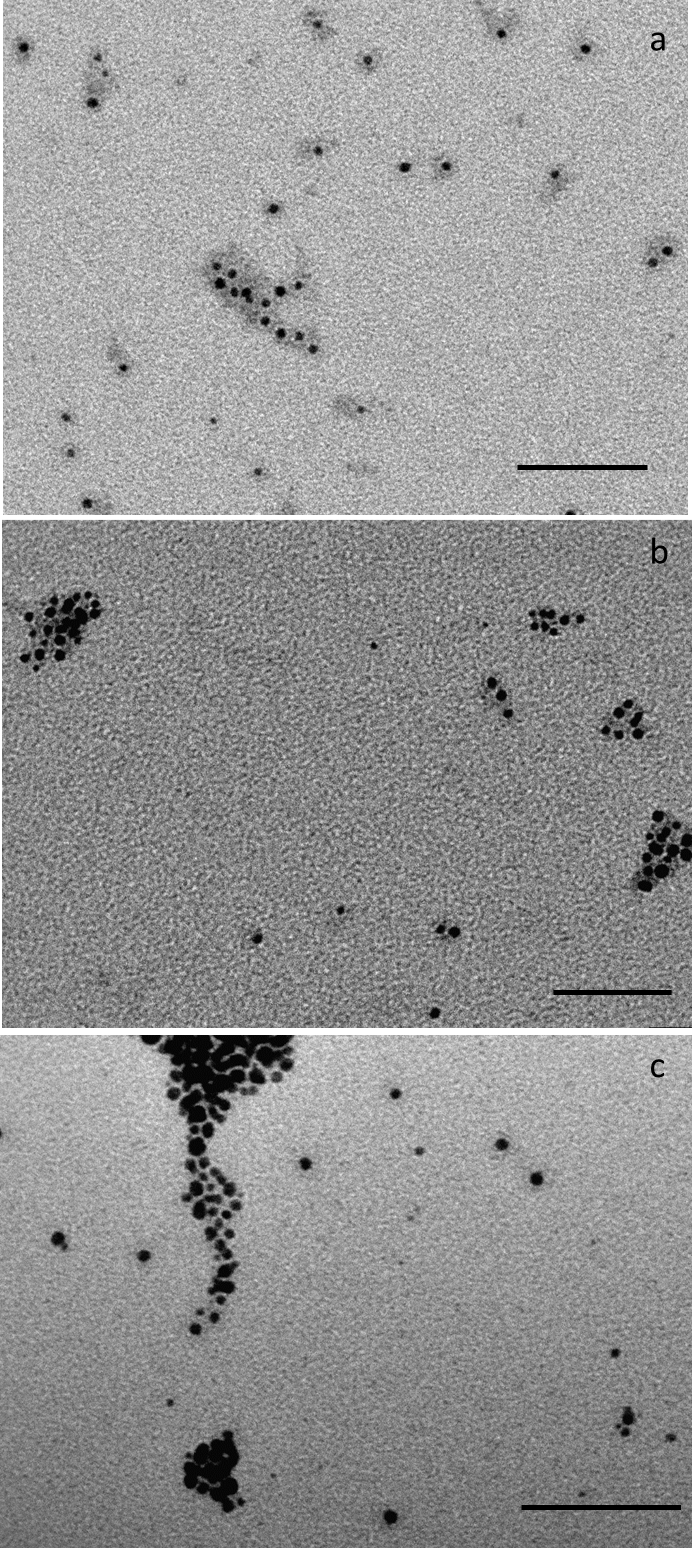


**Figure S9**. TEM micrographs, obtained with staining, of AuNP prepared by Stucky method in presence of Ligand 1, 2 and 4. Scale bar 50 nm.
